# Patients’ Views of Treatment-Focused Genetic Testing (TFGT): Some Lessons for the Mainstreaming of *BRCA1* and *BRCA2* Testing

**DOI:** 10.1007/s10897-018-0261-5

**Published:** 2018-05-11

**Authors:** Sarah Wright, Mary Porteous, Diane Stirling, Julia Lawton, Oliver Young, Charlie Gourley, Nina Hallowell

**Affiliations:** 1 Usher Institute of Population Health Sciences and Informatics, University of Edinburgh, Rm 3.734, Medical School, Teviot Place, Edinburgh, EH8 9AG UK; 2 MRC Institute of Genetics and Molecular Medicine, University of Edinburgh, Edinburgh, UK; 3 Edinburgh Breast Unit, Western General Hospital, Edinburgh, UK; 4 Cancer Research UK Edinburgh Centre, MRC Institute of Genetics and Molecular Medicine, University of Edinburgh, Edinburgh, UK; 5 Big Data Institute, Ethox Centre, Wellcome Centre for Ethics and Humanities, Nuffield Department of Population Health, University of Oxford, Oxford, UK

**Keywords:** Treatment-focused genetic testing (TFGT), *BRCA1* and *BRCA2* testing, Mainstreaming, Breast/ovarian cancer, Clinical implementation, Patient experience

## Abstract

This paper explores patients’ views and experiences of undergoing treatment-focused *BRCA1* and *BRCA2* genetic testing (TFGT), either offered following triaging to clinical genetics (breast cancer) or as part of a mainstreamed care pathway in oncology (ovarian cancer). Drawing on 26 in-depth interviews with patients with breast or ovarian cancer who had undergone TFGT, this retrospective study examines patients’ views of genetic testing at this point in their care pathway, focusing on issues, such as initial response to the offer of testing, motivations for undergoing testing, and views on care pathways. Patients were amenable to the incorporation of TFGT at an early stage in their cancer care irrespective of (any) prior anticipation of having a genetic test or family history. While patients were glad to have been offered TFGT as part of their care, some questioned the logic of the test’s timing in relation to their cancer treatment. Crucially, patients appeared unable to disentangle the treatment role of TFGT from its preventative function for self and other family members, suggesting that some may undergo TFGT to obtain information for others rather than for self.

## Introduction

The cumulative lifetime risk of developing breast cancer associated with a *BRCA1* or *BRCA2* mutation, up to age 80, may be as high as 72% and 69%, respectively (Kuchenbaecker et al. 2017), and approximately 5–10% of newly diagnosed breast cancers are thought to be attributable to a *BRCA1* or *BRCA2* mutation (Claus et al. 1996). For patients already diagnosed with breast cancer, the risk of ipsilateral and contralateral cancers is considered significantly higher among those with a germline BRCA pathogenic variant (Haffty et al. 2002; Kuchenbaecker et al. 2017). Cumulative lifetime risk of ovarian cancer associated with a *BRCA1* or *BRCA2* mutation is estimated at 44% and 17%, respectively (Kuchenbaecker et al. 2017), with 15.5% of patients with epithelial ovarian cancer found to carry a mutation (Zhang et al. 2011). Cancer predisposition genetic testing is offered to patients with breast and/or ovarian cancers (“diagnostic” testing) as well as their unaffected relatives (“predictive” testing). Such tests have allowed for clinical implementation of risk-reducing strategies, including enhanced screening using mammography and or MRI (age-dependent) and risk-reducing surgery (mastectomy, bilateral mastectomy, and/or salpingo-oophorectomy) (National Institute for Health and Care Excellence [NICE] 2013).

Germline BRCA testing has been expanded from its predictive and diagnostic function to inform personalized cancer treatment (NICE 2013); Scottish Intercollegiate Guidelines Network 2013). This decision has been influenced by technological advances in gene sequencing, decreasing costs associated with genetic testing (Trainer et al. 2010) and the results of clinical trials investigating the efficacy of poly(ADP-ribose) polymerase (PARP) inhibitors targeted at BRCA pathogenic variants (George et al. 2017). This new treatment pathway is referred to in a number of ways, including “treatment-focused genetic testing” (hereafter TFGT) and “rapid genetic testing” (NICE 2013). TFGT is offered to newly diagnosed breast and ovarian cancer patients based upon considerations of age at cancer diagnosis, tumor type, and family history. TFGT provides patients and healthcare providers with the information needed to make strategic decisions with regard to definitive surgical interventions and chemotherapeutic regimen while also raising familial awareness of possible hereditary gene mutations.

For breast cancer patients, trials suggest that BRCA status can inform neo-adjuvant chemotherapy, with the use of platinum-based therapy (Telli et al. 2015). When it comes to surgical decision-making, knowledge of a BRCA pathogenic variant can inform the extent of the surgery and the appropriateness of radiotherapy (Weitzel et al. 2003). Since approval by the Food and Drug Administration (FDA), the European Medicines Agency (EMA) (2014), NICE (2016), and the Scottish Medicines Consortium (SMC) (2016), the PARP inhibitor olaparib has been clinically implemented in patients with relapsed, platinum-sensitive high-grade serous ovarian cancer (Fong et al. 2010; Ledermann et al. 2014/Ledermann et al. 2012) for “relapsed and maintenance treatment settings, respectively” (Rafii et al. 2017). With BRCA status providing prognostic information (Candido-dos-Reis et al. 2015) and helping to predict sensitivity to cytotoxic agents (Tan et al. 2008), ovarian cancer management now sees patients “being selected in clinical practice for biomarker-directed therapy, based on presence of a *BRCA1* or *BRCA2* mutation” (George et al. 2017). While PARP inhibitors are now being used in clinical practice for ovarian cancer patients, they have not yet been approved for treatment of breast cancer. In reporting of the Phase 3 OlympiAD trial (ClinicalTrials.gov NCT02000622), Robson et al. provide the latest evidence of the “promising antitumor activity” and progression-free survival associated with Olaparib when compared to standard chemotherapy in breast cancer patients with metastatic disease and a BRCA pathogenic variant (2017). Crucially, findings indicate a need for further investigations, not least examining the “relative efficacy of olaparib and platinum-based chemotherapy” in breast cancer (Robson et al. 2017).

### Mainstreaming Genetic Services

TFGT is symbolic of the shift towards mainstreaming of genetics into cancer care promoted by the UK government (Department of Health 2003; Davies 2017)*.* Mainstreaming, a process that involves the incorporation of genetic services and expertise into standard medical care, represents a “paradigm shift” in the way that healthcare is provided (Rahman 2014). While there are different views as to how mainstreaming should be realized in clinical practice (Kentwell et al. 2017), the over-arching goal remains to offer patients precision medicine and streamlined pathways of care (House of Lords 2009), while also addressing capacity issues faced by specialist genetics services (Slade et al. 2015). In order to achieve universal mainstreamed pathways of care, continued education of health care providers and members of the public is required (Davies 2017; Rahman 2014). At present, while some care pathways are working under a mainstream model, triage and onward referral to genetic services from specialist services continue. This provides an opportunity to compare the experiences of patients in a mainstreamed care pathway with those whose care follows a standard model.

### Patients’ Views of TFGT

While evidence exists as to patients’ experience of diagnostic genetic testing, offered following completion of cancer treatment (Meiser et al. 2008), there is limited evidence relating to how patients view TFGT. Most of this research focuses upon patients who have been offered TFGT during a research study rather than as part of clinical care. Qualitative studies have focused on the information needs and experiences of ovarian cancer patients who underwent TFGT as eligibility criteria for PARP inhibitor trials (Gleeson et al. 2013; Meiser et al. 2012a). Other studies have assessed patients’ information preferences when offered TFGT; trialing streamlined methods of information dissemination (Quinn et al. 2016), comparing patients’ views of written information versus face-to-face communication (Meiser et al. 2012b), and examining timing preferences in relation to treatment decision-making (Wevers et al. 2017). Studies, such as the Genetic Testing in Epithelial Ovarian Cancer (GTEOC) (Plaskocinska et al. 2016), the DNA-BONus study (Høberg-Vetti et al. 2016), and others (Augestad et al. 2017; Meiser et al. 2016; Wevers et al. 2015/Wevers et al. 2016) have examined the acceptability and feasibility of TFGT among participants, in order to inform future roll-out of TFGT in clinical practice. Key findings from this research indicate that TFGT is commonly acceptable to patients who appreciate the treatment implications of timely testing (Gleeson et al. 2013; Meiser et al. 2012a/Meiser et al. 2012b; Wevers et al. 2017). There is little evidence that TFGT is linked to adverse psychological effects (Høberg-Vetti et al. 2016; Wevers et al. 2015/Wevers et al. 2016), although studies have recommended that care should be taken to ensure that patients are treated with care and sensitivity when implementing TFGT (Augestad et al. 2017; Shipman et al. 2017). In summary, research focusing on TFGT suggests that the majority of patients do not find genetic testing to be an additional stress. For those who do, there is an appreciation that undergoing the test brings benefits that outweigh perceived disadvantages (Meiser et al. 2012a).

While numerous studies report the effects of undergoing TFGT in research settings, few have examined patient responses to TFGT when it is offered as routine clinical care to inform surgical interventions or types of adjuvant treatment. In an early, prospective study evaluating the role of “pre-treatment” genetic counseling and testing on surgical decision-making, Schwartz et al. (2005) found that this approach was acceptable to patients, technically feasible, and informed patients’ surgical management. Similarly, in a qualitative study aimed at exploring the attitudes and experiences of women offered TFGT as part of their treatment, Zilliacus et al. (2012) found that TFGT was highly acceptable to patients and there was general consensus that the testing should be part of standard care. Finally, while Wevers et al. (2012) noted that although some women in their study reported experiencing additional distress linked to TFGT, the majority were glad to have been offered the test and recognized its benefits.

While research suggests that patients recognize the benefits of undergoing TFGT, care must be taken when extrapolating from these studies because they involve women who have, in the main, undergone TFGT in research protocols, and this may impact the findings in two ways. First, participants may receive more or different attention and care in research studies than in clinical care, and this may affect their responses. Second, research studies, which include TFGT as an recruitment tool or are specifically looking at the impact of TFGT, may suffer from an ascertainment bias as they may recruit participants who are more positive about TFGT from the outset, because they see study participation as a way of accessing the study drug or gene testing for themselves or their family (McDougall et al. 2016). The study reported circumvents these potential biases by focusing on the experiences of breast and ovarian cancer patients who underwent TFGT as part of their routine clinical care.

## Methods

### Recruitment

Patients were recruited between January and November 2017 from a teaching hospital in the UK. Breast cancer patients were identified by the clinical genetics department. Eligibility criteria used to help identify possible participants included the following: having been offered TFGT; being over 18 years of age; and, being a native English speaker. Furthermore, patients with and without a family history (based on Manchester Score) were approached, as were those with and without an identified BRCA pathogenic variant. In total, 48 patients were sent an information pack which included an “expression of interest” form and pre-paid envelope. Twenty six expression of interest forms were returned, three women subsequently withdrew, due to being unwell, or were subsequently unreachable, and, finally, five women indicated they did not wish to participate.

Ovarian cancer patients received information packs from their oncologist when they presented for check-ups and were given the opportunity to speak to SW in a separate clinic area if they expressed an interest in participation. Eligibility criteria used to help identify possible participants was the same as with the breast cancer patients; however, in addition, consultants were asked to use professional discretion in introducing the study to individual patients, so as to prevent women who were particularly upset during consultation from being approached. In total, 41 ovarian cancer patients were identified as possible participants. SW met with and received expression of interest forms from 12 women. Four women subsequently withdrew from the study, due to being unwell, changing their minds, or not being contactable for interview scheduling.

**Fig. 1:**
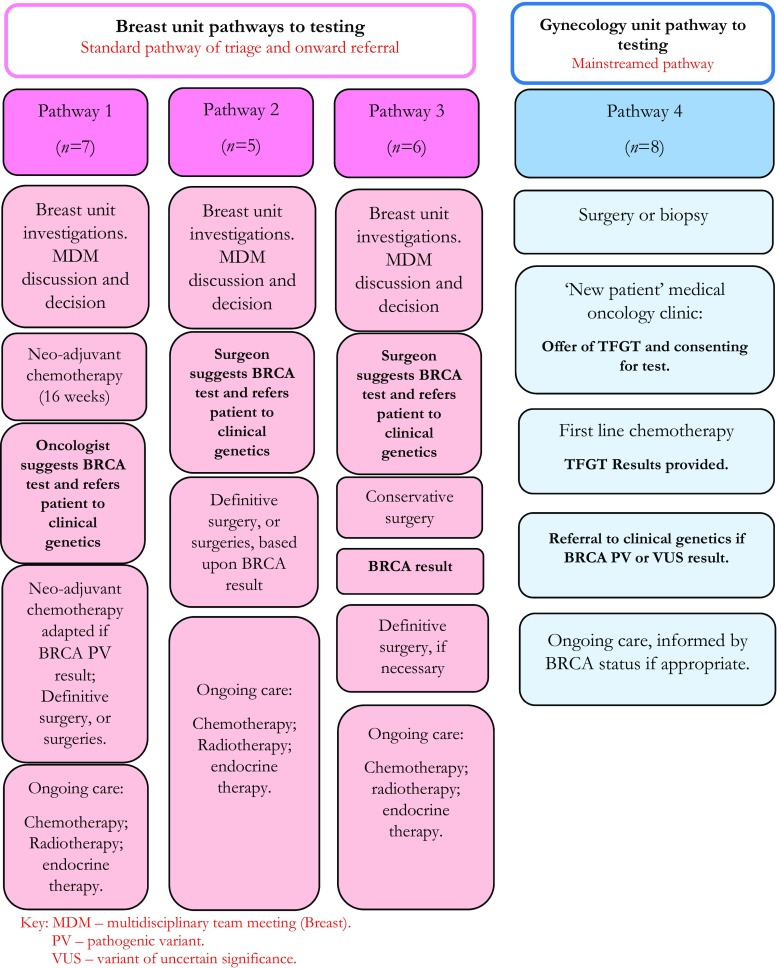
Patients’ pathways to TFGT BRCA testing

All participants underwent TFGT between 2013 and 2017, either as part of a standard pathway involving triage and referral to clinical genetics, in the case of breast cancer patients or, for ovarian cancer patients, as part of their mainstream care in oncology (Fig. 1). Patients were selected so as to include those with a pathogenic variant (hereafter “PV”), those in which no mutation was detected (hereafter “NMD”), and those with a variant of uncertain significance (hereafter “VUS”).

#### TFGT in Breast and Ovarian Cancer Pathways

##### Breast Cancer

For patients with breast cancer, TFGT involved initial triage in the breast clinic with onward referral to clinical genetics for pre-test counseling and testing. The offer of testing was contingent upon consideration of a combination of factors, including the following: age at diagnosis, tumor type, family history, and a discussion about appropriateness of referral at a multidisciplinary team meeting. TFGT was implemented at three different points in the breast treatment pathway, with some patients undergoing conservative surgery (pathway 3) or neoadjuvant therapy (pathway 1) prior to learning of their BRCA result and others undergoing testing prior to any form of cancer treatment (pathway 2) (Fig. 1).

##### Ovarian Cancer

Ovarian cancer patients were consented and tested for a pathogenic BRCA variant during their “new patient” medical oncology appointment (Fig. 1), following surgery, this being as part of the mainstreamed pathway established in 2012. During this consultation, pre-test information was presented by the medical oncologist (Table 1) and supplemented by written documentation from the local clinical genetics team and the charity Target Ovarian Cancer. While no immediate treatment decisions were contingent upon undergoing testing at this stage in the patients’ treatment, genetic test results could inform future treatment options with PARP inhibitors, while also providing family members with knowledge of their own risk.

**Table 1:** **Introducing TFGT to patients**

**Breast unit**	**Gynecology**
Patients are usually triaged for TFGT during their first surgical appointment following case discussion at the weekly MDM. If considered by the team (surgeons and breast oncologists) to meet testing criteria, patients are offered a referral to clinical genetics, by their surgeon, for pre-test counseling and TFGT. The extent to which patients are informed at this stage about the possible implications of TFGT for surgery, adjuvant therapy, and the family varies. For example, it was clear from observations of MDMs and clinician interviews that while some felt comfortable to discuss various implications of TFGT prior to the test, others were more likely to take a staggered approach, that is, only discussing surgical or adjuvant implications if the patient is found to have a PV or VUS at which point the patient is also referred back to clinical genetics for further counseling and family cascading.	Patients are offered TFGT at the “new patient” clinic. The test is usually raised at the end of the consultation and is presented as a straightforward blood test that might help to explain why the individual may have developed cancer. In these appointments, there is little discussion of familial implications, or treatment implications, associated with a PV or VUS, although this varied slightly between consultants. Instead, patients are assured that if the test result comes back with a “positive” result (PV, but also including VUS) that they will be offered an appointment at clinical genetics in order to discuss familial implications with a trained genetic counselor. If the patient wishes to go ahead, a specialist nurse takes the blood following the consultation. Patients are given written information by the consultant to take away with them.

Descriptions based upon observations of MDM, “new patient” oncology clinic, and interviews with clinicians

### Conducting Interviews

The University of Edinburgh Research Ethics Committee approved the study. SW conducted retrospective, semi-structured, face–face interviews with patients, using a broad topic guide, focused on the following: diagnosis of cancer; family history of cancer; expectations and experiences of genetic testing; relevance of TFGT for self and family; views on mainstreaming and/or the timing of testing in relation to cancer diagnosis and opinions about care. Interviews were digitally recorded and lasted between 30 min and 2 h, with the mean for breast cancer patients of 53 min and 57 min for ovarian cancer patients, respectively. Audio files were transcribed verbatim. In addition, SW kept field-notes of observations at the weekly breast multi-disciplinary team meeting and in the “new patient” gynecology clinic. Memoing was used through the fieldwork period in order to facilitate constant reflection and analysis (Maxwell 2012). In order to safeguard participants’ anonymity, pseudonyms were used in all written documentations and are used in the reporting of data.

### Data Analysis

We conducted a thematic analysis of our fieldwork materials (transcripts and field-notes). In the first instance, SW and NH spent time independently familiarizing themselves with the data, listening to audio files, writing up individual patient narratives, and reviewing transcripts (Pope et al. 2000). This process of getting to know our data (Bradley et al. 2007) was the basis of regular meetings between SW and NH, during which time emerging ideas were discussed. Following this exploratory period, SW and NH independently coded all transcripts using NVivo11 software (NVivo qualitative data analysis Software; QSR International Pty Ltd. Version 11, 2015). This step allowed for coding to begin, and, as with early reflections on our data, regular meetings were held between SW and NH in order to ensure consistency in coding and to address any discrepancies. Once all of the data was coded, we started the process of creating larger categories, thus drawing together our data thematically, under three key themes: *The offer of TFGT: Initial reactions*; *Views of care pathways: timing of TFGT in relation to cancer treatment*; and, *Participants’ motivations for undergoing TFGT: constructing hope for prevention.* Our approach to data analysis was inductively and deductively determined (Maxwell 2012), that is, we were influenced both by a priori research questions (for example, seeking to understand patient experiences of undergoing TFGT) as well as recognition of new insights (for example, learning that the treatment function of TFGT is often unacknowledged by patients). In conducting our research, we were informed by an interpretive epistemology that is, concerned with the multitude understandings of the world in which we each find meaning. Further, we position the findings of this research from the ontological standpoint that accepts the “always partial,” contingent nature of knowledge (Denzin 2009).

## Findings/Results

### The Sample

Twenty-six patients, 18 with breast and eight with ovarian cancer, were interviewed (see Table 2). While we had initially planned to interview 15 breast cancer patients and 15 ovarian cancer patients, we found that data saturation was reached after eight interviews with patients with ovarian cancer and felt it suitable to stop recruitment at that point. Of the 26 patients, 20 reported a family history of cancer. Eight were found to carry a BRCA pathogenic variant, 16 had NMD, and two were found to carry a VUS. Despite the fact that BRCA testing is being promoted in newly diagnosed breast cancer patients to inform their surgical decision-making, 6/18 (33%) had already undergone conservative breast surgery (wide local excision), prior to receiving their genetic test result (see Fig. 1, pathway 3). All the ovarian cancer patients had been offered and had undergone genetic testing prior to commencing adjuvant chemotherapy (Fig. 1, pathway 4). Twenty-four participants had undergone TFGT between 6 and 12 months prior to the interviews, while the remaining two patients (1 breast cancer, 1 ovarian cancer) reported undergoing TFGT roughly two years prior to participation in this study. We tried but were unable to recruit patients who declined TFGT.

**Table 2:** Patients with breast cancer and with ovarian cancer

	Patients with breast cancer	Patients with ovarian cancer
Age		
Mean	48	64
Range	33–62	48–82
Marital status		
Married/Partner	15	8
Single	2	–
Divorced	1	–
Children		
Yes	14	8
Yes (adopted/non-biological)	1	–
No	3	–
Employment		
Employed	10	–
Unemployed/not working	4	4
Retired	4	4
Time since cancer diagnosis		
> 2 years	1	1
< 2 years	17	7
Timing of BRCA test		
Prior to any treatment	5	–
During neo-adjuvant chemotherapy	7	–
After wide local excision	6	–
After surgery (ovarian)	–	8
BRCA result		
Pathogenic variant	4	4
No known pathogenic mutation found	12	4
VUS	2	–
Self-reported family history of cancer^a^		
None/none known, + past cancer diagnosis	–	1
None/none known	3	2
≥ 1 first- and second-degree relative (BRCA and/or OVCA)	2	1
≥ 1 first-degree relatives (BRCA and/or OVCA)	7	1
≥ 1 second-degree relatives (BRCA and/or OVCA)	2	1
≥ 1 first-degree relatives (other cancer)	4	2
Anticipated and/or previously asked for genetic test?		
Yes	8	2
No	10	6

^a^For patients from families with extensive family history of different cancers, only the closest relatives have been included. For example, if someone has a grandmother with breast cancer, a grandfather with stomach cancer, and an auntie with breast cancer, they will be listed under ≥ 1 second-degree relatives (BRCA and/or OVCA)

In most instances, cancer type did not appear to have an impact on the experience of being offered TFGT, or perceptions as to the relevance of testing. Instances when differences emerged between the accounts of these groups will be indicated in the succeeding analysis. Three main themes were identified in the data: *The offer of TFGT: Initial reactions*; *Views of care pathways: timing of TFGT in relation to cancer treatment*; and, *Participants’ motivations for undergoing TFGT: constructing hope for prevention.*

### The Offer of TFGT: Initial Reactions

Patients appeared to have different degrees of familiarity with the concept of *BRCA1* and *BRCA2* genetic testing. A number of interviewees were aware of BRCA testing and had, either, sought it out previously or were keen to raise it on this occasion with their clinician. Just over half the women interviewed indicated that, while they had heard of BRCA testing, they had never considered it as relevant to themselves. Finally, only two women stated that the technology was entirely new to them.

A small group described how the offer of TFGT had allowed them to redress previous missed opportunities regarding BRCA testing. These women were generally aware of their family history of cancer and, in some cases, had visited genetic services and requested a genetic test because of their family history. Fran, for example, had previously requested BRCA testing due to her family history of breast cancer only to be refused, as she did not meet testing criteria. This experience of being refused predictive testing appeared to influence Fran’s eagerness in her response to the offer of TFGT:

*I think to myself if they gave me the test when my sister was diagnosed five years before I got my cancer, if they gave me the test … I would never have had cancer* (Breast, PV).

Likewise, Lina described feeling regretful, and angry, when TFGT was raised. She had previously been offered predictive BRCA testing, some two years prior to her diagnosis. She had been keen to proceed at that time, but recalled being advised to go away and think about it. She said, “life took over … I forgot all about it, two years went by. I started feeling not well.”. When she was offered TFGT, her reaction was anger at the missed opportunity to prevent cancer occurring:

*I went to see oncologist and she said, “I think it’s a good idea if we check for the BRCA gene at this point.” Which made me quite angry because I felt that had I had it done when I’d asked for it two years before I could have avoided all of this* (Ovarian, PV)*.*

In Lina’s case, the offer of TFGT was received with complex, and initially negative, emotions. She reflected that her responses had changed with the passing of time, and she described herself as becoming a serious advocate of BRCA testing:

*But yeah, I have, I’ve told everybody that wants to listen … to just go and have a check, you know, especially if they’ve had cancer in the family, I said, “Go and have the BRCA gene [test].” Even my Callanetics teacher! So I do put it out there. I mean it’s available, why not? Why not?* (Ovarian, PV).

While Fran, Lina, and a few others were aware of BRCA testing for reasons linked to their family history of cancer, others’ views of genetic testing appeared to have been influenced and informed by popular culture and the media. For example, Loretta said she had not expected to be offered a BRCA test as part of her care on the basis of her knowledge of BRCA testing, which had been shaped by media discussions of this technology:

*I’d heard of it. I had very much the thought that it was in families where you had you know, mum and aunties and all your siblings and everyone gets breast cancer. … I kind of thought you only hear of these families where everyone has breast cancer or ovarian cancer and that’s the gene. … I didn’t know anything about it in reality* (Breast, PV)*.*

A few of the participants had watched a popular UK soap opera that had a story line based upon BRCA testing and described how this had informed their understanding of the offer of testing and influenced their enthusiastic response:

*When he mentioned that that—the first thing that came into my EastEnders. And I said that to him. I said: I know all about it. I’ve been watching EastEnders. So, aye I didnae [mind]—I just said: aye. Go. Let’s go for it* (Bethany, Ovarian, NMD).

While family history of cancers and/or previous interactions with genetic services appeared to form participants’ initial response to the offer of TFGT, there were a few participants for whom genetic testing seemed a completely novel procedure. Maeve, had no prior awareness of genetic testing, but said she was unperturbed by her oncologist’s offer of TFGT:

*She said, “you don’t have to have it,” you know, she was very, very nice about it and said, you know, “It’s there, the test’s there if you would like to have it.” And I thought it seemed silly not to have it. As I say, it wasn’t anything that was going to be painful or intrusive as far as I was concerned* (Ovarian NMD)*.*

What is clear from participants’ accounts of their initial reaction to the offer of TFGT is that all were keen to undergo testing independent of their cancer type and family history, and irrespective of whether they had previously considered testing before. Yet, while all participants described themselves, in hindsight, as feeling positive about the offer of testing, their views about the timing of testing in relation to other aspects of their treatment were more mixed.

### Views of Care Pathways: Timing of TFGT in Relation to Cancer Treatment

As noted previously (see Fig. 1), the patients in our study underwent BRCA testing at various stages during their care. Breast cancer patients on pathways 1 and 2 were offered, and opted to undergo, TFGT prior to having surgical intervention for their cancer. These patients commonly regarded this sequence of events as logical and timely. For example, Sarah reflected on her experience of being offered the BRCA test while undergoing neo-adjuvant chemotherapy (pathway 1):

*I was pretty fed up when they suggested that it could be the BRCA gene because I mean obviously it’s not a lot of fun being diagnosed with breast cancer and going through chemo and faffing round with everything and then being told you could have the BRCA gene* (Breast, NMD)*.*

Sarah continued, however, saying:

*I mean it’s all very well pussyfooting round it. Say I’d had a lumpectomy and then I had the BRCA gene test and I tested positive then I might then have had to go on and have further treatment [surgery]or chosen to go on and have further treatment [surgery] which I could have had done initially* (Breast, NMD)*.*

Here, Sarah conveys her appreciation of the logic of undergoing TFGT during neo-adjuvant chemotherapy. As she points out, if she had waited to undergo TFGT, she may well have had to undergo multiple treatments (surgeries)—a treatment pathway she clearly views as illogical. Georgina spoke of how she was happy to go ahead with TFGT, when it was raised prior to any form of treatment (pathway 2), seeing it as part of the care available to her and, thus, something to engage with:

*It was just better to keep on going, to be honest. I was quite happy to do every single test underneath the sun for me to be, to find out what was the best option for me to be cancer-free, and what was the quickest way—well, as much as you can go with the NHS to get to that. And once they had suggested the genetic treatment then … again it was just, yeah, I wasn’t anxious at all* (Breast, NMD)*.*

Women who were tested during their neo-adjuvant chemotherapy (pathway 1) or prior to any surgery (pathway 2) commonly regarded the introduction of TFGT as an important and easy step in their care. As hinted at in Sarah’s example, it could be experienced in the short term as an added stress, but this was balanced against an appreciation of the purpose of testing, namely, to determine the most appropriate or logical treatment package for the individual.

Ovarian cancer patients (pathway 4), in contrast, were offered BRCA testing at the “new patient” oncology consultation following their ovarian surgery and prior to commencing adjuvant chemotherapy. Similar to the breast cancer patients on pathways 1 and 2, all of these patients viewed the timing of the test positively, as Maureen indicated:

*No I didnae sort of think, oh my God no, something else, you know. I was quite willing, you know. I don’t know, I think I just, to me it’s just all, it was just partly what I needed to do, kind of thing, you know* (Ovarian, PV)*.*

Not all patients, however, were uniformly positive about the timing of TFGT in relation to their treatment. Unlike pathways 1 and 2, which allowed for the test result to be known prior to *any* breast surgery, pathway 3 saw breast cancer patients undergoing conservative surgery before their BRCA test result had been returned. Proceeding with conservative surgery prior to receiving the BRCA result, only to undergo more radical surgery at a later date, if indicated by TFGT, was regarded by these women as illogical. Indeed, most of these patients said they felt confused by the sequence of events, particularly those who received a positive mutation result after having already undergone conservative breast surgery. While they were pleased to have had their cancer dealt with speedily, this group was left wondering about the logic of enduring multiple surgeries, as Loretta, who had undergone subsequent surgeries, reflected:

*I met with the consultant on the Monday and he said “I’ve got a cancellation on Wednesday. You can come on Wednesday for your operation.” So I had a lumpectomy and my lymph nodes removed. So once that was kind of all done … [he] took me forward for genetic testing. Obviously you kind of think if I had, if somehow the test results could come back instantly, you know then obviously I would have gone for a double mastectomy straight away. And it’s interesting because when I was diagnosed with cancer and [surgeon] said “you can come in on Wednesday and have you know, and have the lumpectomy”, my first response was “can I not have a mastectomy?” So that was my—there was no thought there at all. That just came out of my mouth. And he just said “there is absolutely no need for you to have a mastectomy”. He’s like “the lumpectomy it’s all you need. It’s a very small cancer. That’s far too ridic-—, he’s like, “that’s a ridiculous response” (laughter). In the nicest possible way. But you know there was like we don’t need to go down that road. So obviously part of me thinks ah why didn’t I just push and say, I wanted a mastectomy, because that was obviously my gut reaction* (Breast, PV)*.*

On a more general level, interviewees had differing views of the significance of TFGT in relation to their experience of cancer diagnosis and treatment. For some, TFGT featured prominently, while for others it was only a minor part of their story. In all instances, however, participants felt that TFGT should be made available to others in similar situations. Alice reflected on the impact that TFGT had had on her experience of cancer. For her and her family, the test amounted to a significant moment in her treatment, something that stood out from the multitude of other tests she was undergoing at the time:

*It was quite an important one … Some things stay in your memory and some things go and people have to remind you of them … I can still remember that day, I can still visualize my husband parking the car, going in, sitting at the table, I can still visualize the room, the chair that I sat on to have the bloods taken, it was a really important part of the journey* (Breast, NMD)*.*

For others, TFGT was not a particularly noteworthy event; receiving a diagnosis of cancer, and working through the resulting emotions, meant that genetic testing faded into the background. As Florence said: “*it definitely wasn’t a big upset or anything like that, it was just another thing that was going on at that time.*” Florence went on to say that she “*could probably easily miss it out if I was telling someone the story … it was totally in the shadow of everything else*” (Breast, NMD).

Whether TFGT featured prominently in interviewees’ accounts of their care or not, it was nevertheless the case that, with the exception of some patients in pathway 3, most interviewees approved of the way TFGT had been incorporated into their cancer care. When considering interviewees’ positive response to TFGT, this appeared to have been shaped by their understanding of its relevance, for themselves and family members. Indeed, as the next section indicates, interviewees’ motivations for consenting to TFGT were primarily fueled by their appreciation of the predictive utility of BRCA testing.

### Participants’ Motivations for Undergoing TFGT: Constructing Hope for Prevention

As noted in the “Introduction” section, the purpose of TFGT is to inform decision-making about the treatment of cancer. In the case of breast cancer patients, this relates to, both, neo-adjuvant treatment and surgical options, and in the case of ovarian cancer patients, it relates to chemotherapy regime. While there are clear treatment implications related to a patient’s BRCA status, our findings revealed that appreciation of the individual *treatment* function of TFGT was rarely expressed by breast cancer patients and not at all by ovarian cancer patients. Indeed, for the majority of patients in this study, the main motivation to undergo TFGT was to *prevent future* cancers in self and others, rather than to treat their own cancer.

Breast cancer patients frequently talked about the need to prevent future cancers occurring, and described their uptake of TFGT as motivated by a more general desire to stay alive to parent their children.

*So my main motivation was my children, and really for two reasons. One is I need to survive as long as possible, because at the time [they] were four months, and seven years. And I absolutely want to see them as teenagers. That’s kind of my life goal, I think my main goal in my life is to just see them when they’re, I want both of them to be taller than me one day. And so I wanted to know if I had to have any more operations to minimize the risk* (Pia, Breast, VUS).

For other patients, the motivations to undergo TFGT reflected both a concern to prevent cancer developing in self and determine others’ risks:

*If it had been gene positive, strongly gene positive coming up, then I would have probably been more pushy towards trying to get mastectomies etc. at that stage or at least discuss it further with my breast surgeon as an option as opposed to a lumpectomy so that was where I was coming from. But I’m also aware that while I don’t have children I’ve got two sisters and I’ve got several nieces and nephews and things so it was to really start the creation of a sort of family tree* (Jenny, Breast, NMD).

Finally, there were patients who appeared to be primarily driven to undergo TFGT by their desire to prevent family members from developing cancer by providing them with empowering information. As Alice reflected:

*The whole genetic thing as well it was important for me to know, to have that, to be able to sleep at night knowing right I’ve done that part of it and I’ve done that part of it for my girls. So now my daughters get tested, they’ll get mammograms at 40 rather than 50 so they bring it forward 10 years* (Breast, NMD)*.*

Indeed, the need to act responsibly towards family members was a commonly expressed across both patient groups, with ovarian cancer patients exclusively framing their sole motivation to undergo TFGT as underpinned by the need to generate information for their family:

*I was more concerned that they were carrying it than whether I had it or not, because, well, I wasn’t as young [laughing] as I used to be, and you know, I’d had, I’d got the cancer so, you know … That was, that was my main reason was to see if they were all right, and if they needed to be tested* (Maeve, Ovarian, NMD).

*I would have said, given that I was 67 it wasn’t particularly because of me it was because of my children, and that … yeah, I decided to have it* (Sylvia, Ovarian, PV).

The hope that BRCA testing would garner information that could be mobilized for prevention, coupled with a sense of familial responsibility, featured heavily in all the participants’ accounts. Thus, it was difficult to find patients clearly expressing an understanding of their BRCA test as being treatment-focused, instead it appeared that most interviewees regarded its main role as preventing future cancers in self and others.

## Discussion

Our findings indicate that, irrespective of family history of cancer, cancer type, and care pathway, women were overwhelmingly receptive to TFGT. Furthermore, none of the participants framed TFGT as being too much to take on so soon after their diagnosis. These findings can be examined in relation to the literature concerning the psychological impact of BRCA testing on individuals and confirm that, when conducted with care and with sensitivity to individual needs, BRCA testing is regarded as acceptable (Braithwaite et al. 2004; Meiser et al. 2008; Meiser et al. 2012a; Schlich-Bakker et al. 2006; Wevers et al. 2016; Zilliacus et al. 2012). While the hypothetical idea of BRCA testing post-diagnosis of cancer (Ardern-Jones et al. 2005) or specifically treatment-focused testing (Augestad et al. 2017; Shipman et al. 2017) has been found to raise concerns about, or actual feelings of, heightened emotional distress at a time of being diagnosed with a life-threatening disease, these reservations were not expressed in this study. For some of our participants, TFGT was an attractive option as they already knew about the test and, in a small number of cases, had previously sought it out. Those who had been unable to access testing prior to their cancer diagnosis expressed regret at a missed opportunity—a finding similarly noted in Vadaparampil et al. (2008) in their qualitative study of newly diagnosed breast cancer patients’ views on pre-test genetic counseling. Others’ familiarity with BRCA was shaped by media accounts, and there was evidence of the “Angelina Jolie Effect” (see Evans et al. 2014) informing their (positive) view of the offer of testing. This finding is congruent with what Crabb and LeCouteur (2006) describe as the normalizing effect of popular media representations of (breast) cancer prevention. Even participants for whom TFGT was an entirely novel procedure appeared to trust in the technology, viewing it as beneficial—as something they *should* engage with.

In addition to the positive manner in which participants received the offer of TFGT, the majority felt that the technology was implemented at the right time in their care pathway. Breast cancer patients who awaited their BRCA result prior to starting treatment, or who were undergoing neo-adjuvant chemotherapy at the time of testing, appreciated the logic of the timing of TFGT, specifically in relation to shaping surgical decisions, a finding also noted by Schwartz et al. (2005). Meiser et al. (2012b) report a similar finding in their study of patient preferences for information around breast cancer diagnosis and TFGT. They found that the majority of patients wanted to know of their BRCA status prior to any surgical intervention to ensure that both cancer and risky breast tissue was removed “in one go” (2012). A similar sentiment was echoed by all the breast cancer patients in this study. Indeed, even the patients with a pathogenic variant who underwent conservative surgery prior to learning of their BRCA status, appreciated the impetus to eradicate the cancer, while simultaneously expressing frustration at the illogical sequence of events they had experienced. Other studies examining role and optimum timing of BRCA testing for surgical decision-making have similarly noted discrepancies in the timing of the test and the impact of this on women’s definitive treatment (Stolier et al. 2004; Weitzel et al. 2003). These studies have argued that, ideally, rapid BRCA should be actioned, if possible, to allow women to make best use of simultaneous risk-reducing and therapeutic surgery.

Patients with ovarian cancer were all offered TFGT during their initial oncology consultation, following their surgery, and we found that this timing was acceptable to all. Unlike Shipman et al.’s (2017) study, in which staff observed that undergoing TFGT caused some women additional distress at a time when diagnosis of ovarian cancer had already drained much of their emotional resources, our interviewees reported feeling that BRCA testing was important and demanded little of them. There are a few possible reasons for these differences. First, the observed differences may be due to the fact that Shipman et al. focused on staff perceptions of patients’ experiences, while we focused on patient experiences directly. Second, the patients in our study had had time to come to terms with their diagnosis, which was usually given by their surgeon prior to meeting with their oncologist. By the time they were at their initial oncology consultation, it seems that they were focused on the question of “what to do now?” and open to learn more about the options available to them, including TFGT. Third, while patients in Shipman et al. went through a number of steps to BRCA testing, our patients were consented in one visit, following a brief account of BRCA testing from their oncologist. This may have resulted in them regarding the test as less important and therefore as less anxiety provoking than the patients described by the staff in Shipman et al.’s study, who were informed of both the clinical and familial implications of testing by research genetic counselors prior to TFGT. It must be noted, however, that the breast cancer patients in our study also received information about personal and familial implications of the test in a clinical genetics appointment before undergoing TFGT, but did not appear to respond differently to the ovarian cancer patients we interviewed; suggesting that the timing and extent of information giving in TFGT may not be a contributory factor leading to distress in this patient group as Shipman et al. suggest.

Indeed, our findings suggest that participants’ responses to TFGT might well have been informed by their perception of the test’s utility. We observed in participants’ accounts a common lack of appreciation of the *treatment* function of TFGT, with breast cancer patients rarely understanding the treatment role of the test (Vadaparampil et al. 2008) and ovarian cancer patients appearing to be completely unaware of this. Thus, when it came to the issue of treatment—the element of TFGT that distinguishes it from other forms of germline BRCA testing—there was a general lack of awareness and understanding in participants’ reflections. Not only was immediate treatment of current cancer rarely expressed as a motivator for undergoing TFGT, but in the minority of instances where treatment was understood, it was ambiguously described, and almost indistinguishable from individual’s hopes for prevention. Indeed, patients’ receptiveness to TFGT appeared shaped by their understanding of the test’s preventative utility; most regarded BRCA testing as a means to “weaponize” themselves and their families against future pathological threats—cancer. Thus, these results suggest they conceptualized TFGT as a predictive tool and testing as a familial rather than a personal matter.

It is possible that the lack of recognition regarding the treatment potential of TFGT among participants was due, in part, to the ways in which this technology is mobilized in the clinic. In the case of breast cancer, the treatment or curative response is commonly combined with preventive measures in the form of bilateral mastectomy (Chiba et al. 2016). Thus, it is not surprising that patients might conflate treatment and prevention in their accounts. When it comes to ovarian cancer patients, the treatment role of TFGT is more abstract, as the use of PARP inhibitors for treatment and maintenance only comes in to effect at a later stage, when the patient is relapsed and platinum-sensitive (Alsop et al. 2012). Put simply, the main therapeutic decision contingent upon the genetic test (PARP inhibitor treatment) would not become relevant until relapse. Thus, testing soon after diagnosis for treatment purposes temporally distances the act from its function. With that said, testing at point of diagnosis *does* ensure that patients do not miss the opportunity to have testing, and may save lives in the wider family group as it allows relatives to access risk reducing surgery earlier. With only one of the ovarian cancer patients mentioning being treated with Olaparib at the time of this study (a woman who, it must be noted, did not appear to understand *why* she was on PARPi treatment), it is perhaps unsurprising that these women did not consider that BRCA testing had been undertaken for treatment purposes as it had not resulted in different treatment in their experience.

Although these accounts rarely engaged with the treatment role of TFGT, patients appeared highly motivated to undergo BRCA testing for reasons documented in previous research on predictive and diagnostic testing, namely: to provide information for their family (Douglas et al. 2009; Hallowell 1999; Hallowell et al. 2003; Shipman et al. 2017), to fulfill the gendered role of caring (D’Agincourt-Canning 2001; Rowley 2007), and to ascertain future cancer risks for themselves (Hallowell et al. 2004; Lodder et al. 1999). That these women wished to provide their families with genetic information is not surprising, given that the prognosis for ovarian cancer is often poor (Shipman et al. 2017). Indeed, many of the patients with ovarian cancer in our study regarded themselves as unlikely to benefit personally from preventative steps, but underwent testing for their family’s sake. In this way, our interviewees justified their decision to engage with TFGT not as autonomous individuals but as related and interconnected persons (Hallowell 1999, Hallowell et al. 2003; Rose 2007). As TFGT becomes more widespread in clinical practice it is possible that lay understandings of the treatment role of germline testing will change and patients’ motivations to undergo this form of testing will become more individualistic.

Finally, we suggest that a possible explanation for why the majority of participants in this study appeared to be so positive about the integration of TFGT into their cancer care might be found in what Therond refers to as the “narrative paradox of the BRCA gene” (2016: 46). In her study of mainstreamed BRCA testing for ovarian cancer, Therond observed that patients paradoxically describe BRCA pathogenic variants as both the cause of their suffering and “objects of hope” (2016: 46). This paradox is also present in our interviewees’ accounts, which similarly describe BRCA pathogenic variants as causing cancer and suffering, while also alerting them to, and empowering them (and others) against, future cancer risks.

### Strengths and Limitations

This study contributes to understandings of patients’ views and experiences of TFGT, an area which NICE indicated requires investigation (NICE 2013). Further, the study adds to a paucity of evidence comparing the experiences of TFGT among patients with and without a strong family history of cancer and demonstrated that the presence of a family history of disease has little impact on patients’ views of TFGT. The study is one of the first to compare the experiences of patients undergoing TFGT as part of a standard versus a mainstreamed pathway, indicating that perceptions of the acceptability of TFGT were not influenced by whether testing was offered in specialist or mainstream services.

With regard to its limitations, this study has an overall recruitment rate of 29% (37.5% breast cancer patients, 19.5% ovarian cancer patients). We are aware that the different approaches to recruitment of breast cancer patients (mail-out) and ovarian cancer patients (recruitment through oncologists) might have impacted on recruitment rates. Relying on clinicians to recruit patients directly is not an infallible recruitment method, not least because it depends on them remembering to raise the research with their patients. While the direct approach by clinicians proved the only ethical and practical means for recruiting ovarian cancer patients in this study, it means that we do not have data on the actual number of patients approached, which in turn may mean that our recruitment rates may have exceeded those above. Despite the best of intentions, we were unable to recruit those who had declined TFGT. Anecdotal reports from clinicians involved in the study suggest that nearly all patients approached accept and proceed to testing, and no decliners were identified for recruitment.

As this study was retrospective in design, we do not know to what extent interviewees’ account were tainted by hindsight. Finally, all participants were recruited from the same hospital, albeit through different pathways and at different clinics, and received information (written or face-to-face) about TFGT designed by the same clinical genetics team. It is unclear how or whether this influenced our findings. Indeed, a wide variety of staff were involved in participants’ care and, given that participants’ views recapitulate those of other studies with regard to their experiences of TFGT, it is unlikely that sampling from a single site is problematic.

### Research Recommendations

To the best of our knowledge, we only recruited one patient who recalled taking PARP inhibitors, future research might engage with patients who have undergone PARPi therapy, in order to determine whether this alters their understanding of the treatment role of the test. Finally, future research employing a prospective design, including quantitative methods of data collection, might provide greater insight into changes in perceptions and emotional responses to TFGT offered in the clinical setting and, if multi-sited, may also maximize the chances of recruiting patients who decline the offer of testing.

### Clinical Implications

This study demonstrates that patients with breast or ovarian cancer regard TFGT positively, irrespectively of whether it is offered in mainstream care by a non-genetics specialist or following referral to a specialist genetics clinic. This suggests that mainstreamed genetic testing is acceptable from a patient’s point of view when it is implemented in a supportive manner by trained clinicians. Given that patients often framed their motivations to undergo testing in relation to caring for their family, it is recommended that clinicians should emphasize the individual, *treatment focused* aspects of the test to patients, while nevertheless remaining mindful of, and attending to, the potential informed consent issues pertaining to testing for family members, and the ways in which familial relations work to shape individual decision-making about genetic testing.

This research has demonstrated that patients are comfortable undergoing TFGT in oncology and, thus, provides evidence to support the future mainstreaming of clinical genetics expertise into non-genetic specialties. Through the development of pathways that incorporate genetic services into standard care, the mainstreaming of BRCA testing might help to address some of the capacity issues currently experienced by genetic services in the UK and elsewhere while also providing care that is acceptable to patients.

## References

[CR1] Alsop K, Fereday S, Meldrum C, deFazio A, Emmanuel C, George J, Dobrovic A, Birrer MJ, Webb PM, Stewart C, Friedlander M, Fox S, Bowtell D, Mitchell G (2012). BRCA mutation frequency and patterns of treatment response in BRCA mutation-positive women with ovarian cancer: a report from the Australian Ovarian Cancer Study Group. Journal of Clinical Oncology.

[CR2] ARDERN-JONES A., KENEN R., EELES R. (2005). Too much, too soon? Patients and health professionals' views concerning the impact of genetic testing at the time of breast cancer diagnosis in women under the age of 40. European Journal of Cancer Care.

[CR3] Augestad MT, Høberg-Vetti H, Bjorvatn C, Sekse RJT (2017). Identifying needs: a qualitative study of women’s experiences regarding rapid genetic testing for hereditary breast and ovarian cancer in the DNA BONus study. Journal of Genetic Counseling.

[CR4] Bradley EH, Curry LA, Devers KJ (2007). Qualitative data analysis for health services research: developing taxonomy, themes, and theory. Health Services Research.

[CR5] Braithwaite D, Emery J, Walter F, Prevost AT, Sutton S (2004). Psychological impact of genetic counseling for familial cancer: a systematic review and meta-analysis. Journal of the National Cancer Institute.

[CR6] Candido-dos-Reis FJ, Song H, Goode EL, Cunningham JM, Fridley BL, Larson MC (2015). Germline mutation in BRCA1 or BRCA2 and ten-year survival for women diagnosed with epithelial ovarian cancer. Clinical Cancer Research.

[CR7] Chiba A, Hoskin TL, Hallberg EJ, Cogswell JA, Heins CN, Couch FJ, Boughey JC (2016). Impact that timing of genetic mutation diagnosis has on surgical decision making and outcome for BRCA1/BRCA2 mutation carriers with breast cancer. Annals of Surgical Oncology.

[CR8] Claus EB, Schildkraut JM, Thompson WD, Risch NJ (1996). The genetic attributable risk of breast and ovarian cancer. Cancer.

[CR9] Crabb Shona, LeCouteur Amanda (2006). ‘Fiona Farewells Her Breasts’: A popular magazine account of breast cancer prevention. Critical Public Health.

[CR10] D’Agincourt-Canning L (2001). Experiences of genetic risk: disclosure and the gendering of responsibility. Bioethics.

[CR11] Davies, S.C. “Annual report of the chief medical officer 2016, Generation Genome London: Department of Health (2017).

[CR12] Denzin Norman K. (2009). The elephant in the living room: or extending the conversation about the politics of evidence. Qualitative Research.

[CR13] Department of Health (2003). Our inheritance, our future. Realising the potential of genetics in the NHS. London: The Stationery Office.

[CR14] Douglas HA, Hamilton RJ, Grubs RE (2009). The effect of BRCA gene testing on family relationships: a thematic analysis of qualitative interviews. Journal of Genetic Counseling.

[CR15] Evans DGR, Barwell J, Eccles DM, Collins A, Izatt L, Jacobs C (2014). The Angelina Jolie effect: how high celebrity profile can have a major impact on provision of cancer related services. Breast Cancer Research.

[CR16] Fong PC, Yap TA, Boss DS, Carden CP, Mergui-Roelvink M, Gourley C, de Greve J, Lubinski J, Shanley S, Messiou C, A'Hern R, Tutt A, Ashworth A, Stone J, Carmichael J, Schellens JHM, de Bono JS, Kaye SB (2010). Poly (ADP)-ribose polymerase inhibition: frequent durable responses in BRCA carrier ovarian cancer correlating with platinum-free interval. Journal of Clinical Oncology.

[CR17] George A, Kaye S, Banerjee S (2017). Delivering widespread BRCA testing and PARP inhibition to patients with ovarian cancer. Nature Reviews Clinical Oncology.

[CR18] Gleeson M, Meiser B, Barlow-Stewart K, Trainer AH, Tucker K, Watts KJ, Friedlander M, Kasparian N (2013). Communication and information needs of women diagnosed with ovarian cancer regarding treatment-focused genetic testing. In Oncology Nursing Forum.

[CR19] Haffty BG, Harrold E, Khan AJ, Pathare P, Smith TE, Turner BC, Glazer PM, Ward B, Carter D, Matloff E, Bale AE, Alvarez-Franco M (2002). Outcome of conservatively managed early-onset breast cancer by BRCA1/2 status. The Lancet.

[CR20] Hallowell N (1999). Doing the right thing: genetic risk and responsibility. Sociology of Health & Illness.

[CR21] Hallowell N, Foster C, Eeles R, Ardern-Jones A, Murday V, Watson M (2003). Balancing autonomy and responsibility: the ethics of generating and disclosing genetic information. Journal of Medical Ethics.

[CR22] Hallowell N, Foster C, Eeles R, Ardern-Jones A, Watson M (2004). Accommodating risk: responses to BRCA1/2 genetic testing of women who have had cancer. Social Science & Medicine.

[CR23] Høberg-Vetti H, Bjorvatn C, Fiane BE, Aas T, Woie K, Espelid H, Rusken T, Eikesdal HP, Listøl W, Haavind MT, Knappskog PM, Haukanes BI, Steen VM, Hoogerbrugge N (2016). BRCA1/2 testing in newly diagnosed breast and ovarian cancer patients without prior genetic counselling: the DNA-BONus study. European Journal of Human Genetics.

[CR24] House of Lords. (2009). Genomic medicine*.* (HL Paper 107-I). London: The Stationary Office.

[CR25] Kentwell M, Dow E, Antill Y, Wrede CD, McNally O, Higgs E, Hamilton A, Ananda S, Lindeman GJ, Scott CL (2017). Mainstreaming cancer genetics: a model integrating germline BRCA testing into routine ovarian cancer clinics. Gynecologic Oncology.

[CR26] Kuchenbaecker KB, Hopper JL, Barnes DR, Phillips KA, Mooij TM, Roos-Blom MJ, Jervis S, van Leeuwen FE, Milne RL, Andrieu N, Goldgar DE, Terry MB, Rookus MA, Easton DF, Antoniou AC, McGuffog L, Evans DG, Barrowdale D, Frost D, Adlard J, Ong KR, Izatt L, Tischkowitz M, Eeles R, Davidson R, Hodgson S, Ellis S, Nogues C, Lasset C, Stoppa-Lyonnet D, Fricker JP, Faivre L, Berthet P, Hooning MJ, van der Kolk LE, Kets CM, Adank MA, John EM, Chung WK, Andrulis IL, Southey M, Daly MB, Buys SS, Osorio A, Engel C, Kast K, Schmutzler RK, Caldes T, Jakubowska A, Simard J, Friedlander ML, McLachlan SA, Machackova E, Foretova L, Tan YY, Singer CF, Olah E, Gerdes AM, Arver B, Olsson H, and the BRCA1 and BRCA2 Cohort Consortium (2017). Risks of breast, ovarian, and contralateral breast cancer for BRCA1 and BRCA2 mutation carriers. JAMA.

[CR27] Ledermann J, Harter P, Gourley C, Friedlander M, Vergote I, Rustin G, Scott C, Meier W, Shapira-Frommer R, Safra T, Matei D, Macpherson E, Watkins C, Carmichael J, Matulonis U (2012). Olaparib maintenance therapy in platinum-sensitive relapsed ovarian cancer. New England Journal of Medicine.

[CR28] Ledermann J, Harter P, Gourley C, Friedlander M, Vergote I, Rustin G, Scott CL, Meier W, Shapira-Frommer R, Safra T, Matei D, Fielding A, Spencer S, Dougherty B, Orr M, Hodgson D, Barrett JC, Matulonis U (2014). Olaparib maintenance therapy in patients with platinum-sensitive relapsed serous ovarian cancer: a preplanned retrospective analysis of outcomes by BRCA status in a randomised phase 2 trial. The Lancet Oncology.

[CR29] Lodder LN, Frets PG, Trijsburg RW, Meijers-Heijboer EJ, Klijn JGM, Duivenvoorden HJ (1999). Presymptomatic testing for BRCA1 andBRCA2: how distressing are the pre-test weeks?. Journal of Medical Genetics.

[CR30] Maxwell, J. A. (2012). Qualitative research design: an interactive approach (Vol. 41). Sage publications.

[CR31] McDougall, R., Martin, D., Gillam, L., Hallowell, N., Brookes, A., & Guillemin, M. (2016). Therapeutic appropriation: a new concept in the ethics of clinical research. *Journal of Medical Ethics*, medethics-2016. 10.1136/medethics-2016-103612 27733438

[CR32] Meiser B, Saunders C, Lobb E, Mitchell G, Tucker K, Barlow-Stewart K (2008). Genetic counselling and testing for inherited gene mutations in newly diagnosed patients with breast cancer: a review of the existing literature and a proposed research agenda. Breast Cancer Research.

[CR33] Meiser B, Gleeson M, Kasparian N, Barlow-Stewart K, Ryan M, Watts K, Menon D, Mitchell G, Tucker K (2012). There is no decision to make: experiences and attitudes toward treatment-focused genetic testing among women diagnosed with ovarian cancer. Gynecologic Oncology.

[CR34] Meiser B, Gleeson M, Watts K, Peate M, Zilliacus E, Barlow-Stewart K, Saunders C, Mitchell G, Kirk J (2012). Getting to the point: what women newly diagnosed with breast cancer want to know about treatment-focused genetic testing. Oncology Nursing Forum.

[CR35] Meiser B, Quinn VF, Gleeson M, Kirk J, Tucker KM, Rahman B, Saunders C, Watts KJ, Peate M, Geelhoed E, Barlow-Stewart K, Field M, Harris M, Antill YC, Mitchell G (2016). When knowledge of a heritable gene mutation comes out of the blue: treatment-focused genetic testing in women newly diagnosed with breast cancer. European Journal of Human Genetics.

[CR36] National Institute for Health and Care Excellence. (2016). Olaparib for maintenance treatment of relapsed, platinum-sensitive, BRCA mutation-positive ovarian, fallopian tube and peritoneal cancer after response to second-line or subsequent platinum-based chemotherapy. NICE technology appraisal guidance [TA381].

[CR37] National Institute for Health and Clinical Excellence (2013). Familial breast cancer: classification, care and managing breast cancer and related risks in people with a family history of breast cancer. NICE guideline [CG164]. 31940157

[CR38] Plaskocinska, I., Shipman, H., Drummond, J., Thompson, E., Buchanan, V., Newcombe, B., et al. (2016). New paradigms for BRCA1/BRCA2 testing in women with ovarian cancer: results of the Genetic Testing in Epithelial Ovarian Cancer (GTEOC) study. Journal of Medical Genetics, jmedgenet-2016. 10.1136/jmedgenet-2016-103902 PMC5099175 27208206

[CR39] Pope C, Ziebland S, Mays N (2000). Qualitative research in health care: analysing qualitative data. BMJ: British Medical Journal.

[CR40] Quinn VF, Meiser B, Kirk J, Tucker KM, Watts KJ, Rahman B, Peate M, Saunders C, Geelhoed E, Gleeson M, Barlow-Stewart K, Field M, Harris M, Antill YC, Cicciarelli L, Crowe K, Bowen MT, Mitchell G (2016). Streamlined genetic education is effective in preparing women newly diagnosed with breast cancer for decision making about treatment-focused genetic testing: A randomized controlled noninferiority trial. Genetics in Medicine.

[CR41] Rafii S, Gourley C, Kumar R, Geuna E, Ang JE, Rye T (2017). Baseline clinical predictors of antitumor response to the PARP inhibitor olaparib in germline BRCA1/2 mutated patients with advanced ovarian cancer. Oncotarget.

[CR42] Rahman N (2014). Mainstreaming genetic testing of cancer predisposition genes. Clinical Medicine.

[CR43] Robson M, Im SA, Senkus E, Xu B, Domchek SM, Masuda N, Delaloge S, Li W, Tung N, Armstrong A, Wu W, Goessl C, Runswick S, Conte P (2017). Olaparib for metastatic breast cancer in patients with a germline BRCA mutation. New England Journal of Medicine.

[CR44] Rose, N. (2007). The politics of life itself: biomedicine. Power, and subjectivity in the twenty-first century. Princeton University Press.

[CR45] Rowley E (2007). On doing ‘being ordinary’: women’s accounts of BRCA testing and maternal responsibility. New Genetics and Society.

[CR46] Schlich-Bakker Kathryn J., Wárlám-Rodenhuis Carla C., van Echtelt Jeanne, van den Bout Jan, Ausems Margreet G.E.M., ten Kroode Herman F.J. (2006). Short term psychological distress in patients actively approached for genetic counselling after diagnosis of breast cancer. European Journal of Cancer.

[CR47] Schwartz MD, Lerman C, Brogan B, Peshkin BN, Isaacs C, DeMarco T (2005). Utilization of BRCA1/BRCA2 mutation testing in newly diagnosed breast cancer patients. Cancer Epidemiology and Prevention Biomarkers.

[CR48] Scottish Intercollegiate Guidelines Network. Management of epithelial ovarian cancer*.* Edinburgh: SIGN; 2013.

[CR49] Shipman H, Flynn S, MacDonald-Smith CF, Brenton J, Crawford R, Tischkowitz M (2017). Universal BRCA1/BRCA2 testing for ovarian cancer patients is welcomed, but with care: how women and staff contextualize experiences of expanded access. Journal of Genetic Counseling.

[CR50] Slade I, Riddell D, Turnbull C, Hanson H, Rahman N (2015). Development of cancer genetic services in the UK: a national consultation. Genome Medicine.

[CR51] Stolier AJ, Fuhrman GM, Mauterer L, Bolton JS, Superneau DW (2004). Initial experience with surgical treatment planning in the newly diagnosed breast cancer patient at high risk for BRCA-1 or BRCA-2 mutation. The Breast Journal.

[CR52] Tan DS, Rothermundt C, Thomas K, Bancroft E, Eeles R, Shanley S (2008). “BRCAness” syndrome in ovarian cancer: a case–control study describing the clinical features and outcome of patients with epithelial ovarian cancer associated with BRCA1 and BRCA2 mutations. Journal of Clinical Oncology.

[CR53] Telli ML, Jensen KC, Vinayak S, Kurian AW, Lipson JA, Flaherty PJ, Timms K, Abkevich V, Schackmann EA, Wapnir IL, Carlson RW, Chang PJ, Sparano JA, Head B, Goldstein LJ, Haley B, Dakhil SR, Reid JE, Hartman AR, Manola J, Ford JM (2015). Phase II study of gemcitabine, carboplatin, and iniparib as neoadjuvant therapy for triple-negative and BRCA1/2 mutation-associated breast cancer with assessment of a tumor-based measure of genomic instability: PrECOG 0105. Journal of Clinical Oncology.

[CR54] Therond, C (2016) ‘The changing nature of genetic testing: mainstreaming BRCA genetic testing in ovarian cancer in the practice of oncology’, MSc in Medical Anthropology, UCL, London, UK.

[CR55] Trainer AH, Lewis CR, Tucker K, Meiser B, Friedlander M, Ward RL (2010). The role of BRCA mutation testing in determining breast cancer therapy. Nature Reviews Clinical Oncology.

[CR56] Vadaparampil ST, Quinn GP, Brzosowicz JP, Miree CA (2008). Experiences of genetic counseling for BRCA1/2 among recently diagnosed breast cancer patients: a qualitative inquiry. Journal of Psychosocial Oncology.

[CR57] Weitzel JN, McCaffrey SM, Nedelcu R, MacDonald DJ, Blazer KR, Cullinane CA (2003). Effect of genetic cancer risk assessment on surgical decisions at breast cancer diagnosis. Archives of Surgery.

[CR58] Wevers MR, Hahn DE, Verhoef S, Bolhaar MD, Ausems MG, Aaronson (2012). Breast cancer genetic counseling after diagnosis but before treatment: a pilot study on treatment consequences and psychological impact. Patient Education and Counseling.

[CR59] Wevers MR, Schmidt MK, Engelhardt EG, Verhoef S, Hooning MJ, Kriege M, Seynaeve C, Collée M, van Asperen CJ, Tollenaar RAEM, Koppert LB, Witkamp AJ, Rutgers EJT, Aaronson NK, Rookus MA, Ausems MGEM (2015). Timing of risk reducing mastectomy in breast cancer patients carrying a BRCA1/2 mutation: retrospective data from the Dutch HEBON study. Familial Cancer.

[CR60] Wevers MR, Ausems MG, Verhoef S, Bleiker EM, Hahn DE, Brouwer T (2016). Does rapid genetic counseling and testing in newly diagnosed breast cancer patients cause additional psychosocial distress? Results from a randomized clinical trial. Genetics in Medicine.

[CR61] Wevers MR, Aaronson NK, Bleiker E, Hahn DE, Brouwer T, van Dalen T (2017). Rapid genetic counseling and testing in newly diagnosed breast cancer: patients’ and health professionals’ attitudes, experiences, and evaluation of effects on treatment decision making. Journal of Surgical Oncology.

[CR62] Zhang S, Royer R, Li S, McLaughlin JR, Rosen B, Risch HA, Fan I, Bradley L, Shaw PA, Narod SA (2011). Frequencies of BRCA1 and BRCA2 mutations among 1,342 unselected patients with invasive ovarian cancer. Gynecologic Oncology.

[CR63] Zilliacus E, Meiser B, Gleeson M, Watts K, Tucker K, Lobb EA, Mitchell G (2012). Are we being overly cautious? A qualitative inquiry into the experiences and perceptions of treatment-focused germline BRCA genetic testing amongst women recently diagnosed with breast cancer. Supportive Care in Cancer.

